# Notch signaling regulates strain-mediated phenotypic switching of vascular smooth muscle cells

**DOI:** 10.3389/fcell.2022.910503

**Published:** 2022-08-12

**Authors:** Cansu Karakaya, Mark C. van Turnhout, Valery L. Visser, Tommaso Ristori, Carlijn V. C. Bouten, Cecilia M. Sahlgren, Sandra Loerakker

**Affiliations:** ^1^ Department of Biomedical Engineering, Eindhoven University of Technology, Eindhoven, Netherlands; ^2^ Institute for Complex Molecular Systems, Eindhoven University of Technology, Eindhoven, Netherlands; ^3^ Faculty of Science and Engineering, Åbo Akademi University, Turku, Finland

**Keywords:** Notch signaling, vascular smooth muscle cells, phenotype, mechanosensitive, strain, stretch quantification, cardiovascular

## Abstract

Mechanical stimuli experienced by vascular smooth muscle cells (VSMCs) and mechanosensitive Notch signaling are important regulators of vascular growth and remodeling. However, the interplay between mechanical cues and Notch signaling, and its contribution to regulate the VSMC phenotype are still unclear. Here, we investigated the role of Notch signaling in regulating strain-mediated changes in VSMC phenotype. Synthetic and contractile VSMCs were cyclically stretched for 48 h to determine the temporal changes in phenotypic features. Different magnitudes of strain were applied to investigate its effect on Notch mechanosensitivity and the phenotypic regulation of VSMCs. In addition, Notch signaling was inhibited *via* DAPT treatment and activated with immobilized Jagged1 ligands to understand the role of Notch on strain-mediated phenotypic changes of VSMCs. Our data demonstrate that cyclic strain induces a decrease in Notch signaling along with a loss of VSMC contractile features. Accordingly, the activation of Notch signaling during cyclic stretching partially rescued the contractile features of VSMCs. These findings demonstrate that Notch signaling has an important role in regulating strain-mediated phenotypic switching of VSMCs.

## Introduction

Tissue growth and remodeling are mainly mediated by cells, and mechanical cues play a pivotal role in this process (Humphrey, 2008; Han et al., 2018; Ambrosi et al., 2019). In the vessel wall, vascular smooth muscle cells (VSMCs) are important regulators of vascular remodeling, given their ability to express different phenotypes (Owens et al., 2004; Rensen et al., 2007). VSMCs display a quiescent contractile phenotype in the homeostatic state, but are able to switch to a synthetic phenotype upon changes in hemodynamic conditions to induce growth and remodeling. Although cyclic strain experienced by VSMCs is known to affect their phenotype, both up and downregulation of contractile phenotype markers upon cyclic strain compared to static controls have been reported (Reusch et al., 1996; Tock et al., 2003; Butcher et al., 2006; Hu et al., 2014; Yao et al., 2014; Rodríguez et al., 2015; Wan et al., 2015). In addition, the underlying mechanisms of strain-mediated changes in VSMC phenotype have not been fully unraveled. Understanding the mechanobiological mechanisms regulating VSMC fate is essential to unravel the processes characterizing healthy vascular development and pathogenesis, as well as to realize the growth and remodeling of tissue-engineered vascular grafts, which have not always demonstrated native-like tissue organization and physiological long-term functionality (Tara et al., 2015; Khosravi et al., 2016; Sugiura et al., 2016, Sugiura et al.,2017; Yang et al., 2016; Duijvelshoff et al., 2021). It also may be important for controlling vascular development and remodeling, and ultimately obtaining functional tissue-engineered vascular grafts.

Mechano-regulated cell signaling pathways may explain the link between mechanical cues and cell behavior regulating growth and remodeling (Karakaya et al., 2022). In particular, the phenotypic plasticity of VSMCs is modulated by several signaling pathways such as transforming growth factor beta (Hautmann et al., 1997; Adam et al., 2000), Hippo/yes-associated protein (Wang X. et al., 2012; Xie et al., 2012; Kimura et al., 2016), and Notch (Domenga et al., 2004; Doi et al., 2006; Noseda et al., 2006; High et al., 2007; Lin and Lilly, 2014b), which are increasingly recognized as being mechanosensitive (Mata-Greenwood et al., 2003, Mata-Greenwood et al., 2005; Morrow et al., 2005; Baker et al., 2008; Codelia et al., 2014; Wang et al., 2016; Mack et al., 2017; Driessen et al., 2018; Loerakker et al., 2018; Dong et al., 2019; van Engeland et al., 2019; Yamashiro et al., 2020; Stassen et al., 2021). In this study, our focus is on the mechanosensitive Notch signaling pathway, which plays a role in the differentiation of VSMCs and the maintenance of the contractile phenotype (High et al., 2008; Liu et al., 2009). In addition, Notch signaling is known to be involved in development, remodeling, and homeostasis of the vasculature (Iso et al., 2003; Gridley, 2007). Notch is a contact-dependent signaling pathway where a ligand in one cell binds to and activates the Notch receptor in an adjacent cell. In the vessel wall, Jagged1 ligands expressed by the endothelial cells bind to the Notch receptor of the neighboring VSMCs and activate the Notch signaling in a feed-forward manner. This interaction enables Notch signaling to propagate through the VSMC layers *via* a process called lateral induction and promotes the differentiation of VSMCs (Hoglund and Majesky, 2012; Manderfield et al., 2012; Baeten and Lilly, 2017). Yet, how and to what extent the interplay between strain and Notch signaling affects the VSMC phenotype in the context of vascular remodeling has not been investigated. Given the important role of Notch in regulating vascular development and adaptation (Iso et al., 2003; Gridley, 2007), we hypothesize that Notch signaling can directly regulate the VSMC phenotype in response to mechanical stress. For contractile, homeostatic VSMCs, we expect that an increase in strain experienced by VSMCs leads to a decrease in Notch signaling between VSMCs and, subsequently, a (partial) switch from the contractile to the synthetic phenotype. This would enable growth and remodeling in the case of increased hemodynamic loading.

To test this hypothesis *in vitro*, we obtained synthetic and contractile phenotypes of VSMCs, and cyclically stretched them for 48 h to determine changes in their phenotypic markers due to strain. To understand the direct effect of Notch signaling on the strain-mediated phenotypic regulation of VSMCs, we inhibited Notch signaling by the γ-secretase inhibitor DAPT treatment and activated Notch *via* immobilizing Jagged1 ligands at the cell-substrate in static and stretched samples. Jagged1 ligands were used to mimic the endothelial cell signal that activates Notch signaling in VSMCs. In addition, the magnitude of applied strain was varied to investigate the effect of different magnitudes of strain on Notch mechanosensitivity and on the regulation of VSMC phenotype. Our results showed that the application of strain to contractile VSMCs resulted in a transition towards the synthetic phenotype. Notch signaling decreased in strained VSMCs, in line with the loss of their contractile features. The inhibition of Notch signaling *via* DAPT in stretched contractile VSMCs did not have additional effect in that setting. In contrast, the activation of Notch signaling by immobilized Jagged1 ligands during stretching partially rescued the contractile features, thereby demonstrating that Notch signaling has an important role in regulating strain-mediated changes in VSMC phenotype.

## Materials and methods

### Cell culture

Human coronary artery smooth muscle cells (Lonza) were cultured in Human Vascular Smooth Muscle Cell Basal Medium (Gibco) supplemented with 1% penicillin/streptomycin (Sigma-Aldrich) and either 5% Smooth Muscle Growth Supplement (SMGS, Gibco) to obtain synthetic VSMCs, or 1% Smooth Muscle Differentiation Supplement (SMDS, Gibco) for a minimum of 7 days to obtain contractile VSMCs. Cells were cultured in a humidified incubator at 37°C with 5% CO_2_, and media was refreshed every 2–3 days. Cells were passaged when 80%–90% confluent and used in experiments at passage 6 to 8.

### Activation and inhibition of Notch signaling

Bioflex culture plates (untreated, Flexcell Int.) were coated with 2.2 µg/cm^2^ of bovine fibronectin (Thermo Fisher Scientific) on 4 cm^2^ of the center of the wells for 1 h at 37°C. The wells were treated with 1% Pluronic F-127 (Sigma-Aldrich) for 5 min at room temperature to prevent aspecific adhesions. For the induction of Notch signaling, 50 μg/ml Recombinant Protein G (Thermo Fisher Scientific) in PBS were added to the fibronectin-coated membranes and incubated overnight at room temperature. Plates were incubated with 2 μg/ml of Recombinant Human Jagged1-Fc Chimera Protein (R and D Systems) in 0.1% bovine serum albumin (BSA)/PBS for 3 h at room temperature. Cells were immediately seeded on Bioflex culture plates with densities of 100.000 cells/4 cm^2^ for synthetic VSMCs and 150.000 cells/4 cm^2^ for contractile VSMCs and left to attach overnight. Notch inhibition was performed by adding 10 μg/ml γ-secretase inhibitor DAPT (Sigma- Aldrich) to the culture media from stock solutions in sterile dimethyl sulfoxide (DMSO) (Sigma-Aldrich). The same concentration of the vehicle DMSO was added to the other samples for comparison. DMSO/DAPT was refreshed every 24 h.

### Stretch experiments and quantification

**FIGURE 1:**
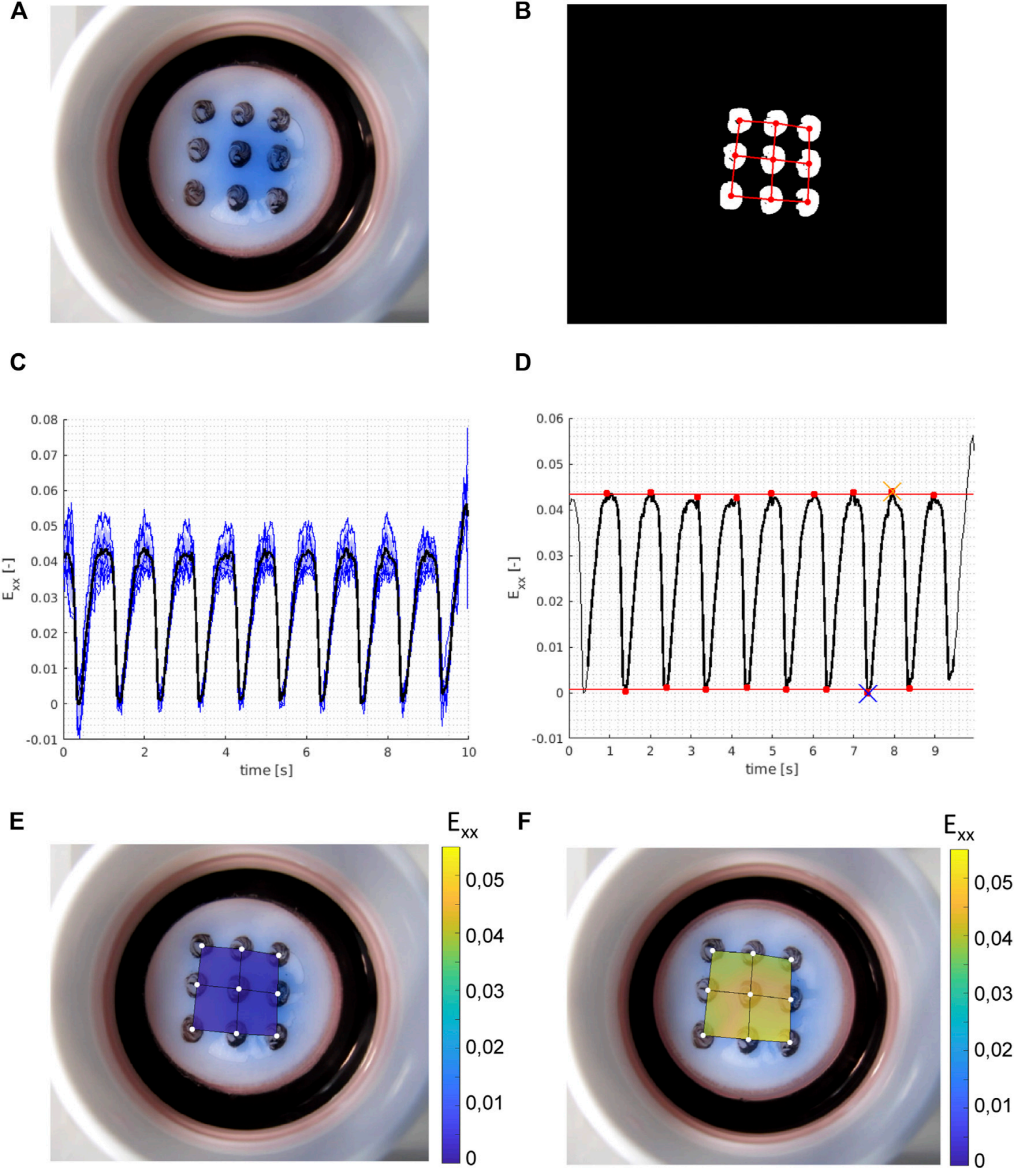
Example of experimental strain analysis. With **(A)** a raw (RGB) frame from the movie showing the Bioflex plate and the dot pattern; **(B)** binary frame after image pre-processing with the isolated grid dots and with the finite element mesh super-imposed in red; **(C)** calculated (horizontal) strains (E_xx_) in all nine grid dots over time (blue, thin) with the mean strain (black, thick) over time; **(D)** peak analysis on the time averaged strains (black, thick) from **(C)** with the identified peaks and valleys (red dots), and the mean valley strains (bottom red line) and mean peak strains (top red line) which give us the calculated horizontal strain amplitude of the sample. The blue cross shows the reference frame for the valley strain visualized in **(E)** and the orange cross shows the reference frame for the peak strain visualized in **(F)**. Vertical strain amplitudes are calculated with the same method, and the average of horizontal and vertical strain amplitudes is taken as the approximate equibiaxial strain level of the sample.

The Bioflex culture plates were mounted on circular 25-mm posts and equibiaxially stretched with the FX-5000™ Tension System (Flexcell Int.) with different strain magnitudes at 1 Hz for 48 h. Unstrained samples of synthetic and contractile VSMCs cultured under identical conditions as the strained samples were used as controls. Different levels of cyclic strain were applied on the dynamically stretched samples. For quantification of these different levels of cyclic strain, a custom-built script in MATLAB (MathWorks) was used. Briefly, nine locations at the bottom of the flexible membranes were marked and traced live with a camera (Canon Powershot G16) to quantify the displacement of each location (Figure 1A). The movie frames were pre-processed into binary images with custom written MATLAB scripts (https://gitlab.tue.nl/stem/dgslab), and the location of the centers of the dots was traced over time (Figure 1B). The dot pattern was converted to a linear finite element mesh (Oomens et al., 2018) and we used an in-house MATLAB finite element program (https://gitlab.tue.nl/stem/mlfem_nac) to calculate the linear strains at the positions of the dots in each frame. The horizontal (xx) strains of all dots were averaged in each frame (Figure 1C), and the resulting two time-dependent average strain patterns were subjected to a peak analysis with the MATLAB function *findpeaks* (Figure 1D). We calculated the horizontal (xx) strain amplitudes in the movie (sample) as the difference of the mean peak strains and mean valley strains (Figures 1E,F). Vertical (yy) strain amplitudes were calculated with the same method. Finally, the average amplitude of horizontal (xx) and vertical (yy) strains was taken as the approximate equibiaxial strain level that was applied onto the membrane. This analysis was performed for each flexible membrane, to quantify different strain levels applied to the samples.

### Immunofluorescence staining

Upon completion of the stretching experiments, the samples were fixed with 3.7% formaldehyde for 20 min at room temperature, and permeabilized with 0.5% Triton X-100 in PBS for 3 min. Non-specific antibody binding was blocked 30 min with 3% BSA in PBS. The samples were subsequently incubated with primary antibodies in 1.5% BSA in PBST at 4°C overnight. The primary antibodies used in this study were alpha smooth muscle actin (αSMA) (A2547, Sigma-Aldrich, 1:600), KI67 (PA5-16446, Invitrogen, 1:100) and collagen I (C2456, Sigma-Aldrich, 1:100). The samples were washed three times for 5 min with PBST and incubated with secondary antibodies in PBST for 1 h at room temperature. The secondary antibodies used were goat anti-mouse IgG2a Alexa Fluor 488 (A21131, Invitrogen, 1:1,000), goat anti-rabbit IgG Alexa Fluor 555 (A21428, Invitrogen, 1:500) and goat anti-mouse IgG1 Alexa Fluor 488 (A21121, Invitrogen, 1:500). All samples were stained for DAPI (Sigma-Aldrich, 1:500) to visualize cell nuclei. Subsequently, the samples were mounted with Mowiol (Sigma-Aldrich) on thin coverslips. Image acquisition was done using an inverted fluorescence microscope (Leica DMi8) with ×20 magnification. Negative controls of immunofluorescence (IF) staining are presented in Supplementary Figure S1.

### qPCR analysis

RNA was isolated using the RNeasy mini kit (74106, Qiagen) according to the manufacturer’s protocol, and a 15 min DNase incubation (79256, Qiagen) was performed to exclude genomic DNA. The concentration and purity of the isolated RNA were measured with the Nanodrop 1,000 (Thermo Fisher Scientific). cDNA was synthetized in a thermal cycler (C1000 Touch, Bio-Rad) with a reaction containing 165 ng of RNA, 1 μl of 50 ng/μl random primers (Promega), 1 μl of 10 mM dNTPs (Invitrogen), 4 μl of 5x first strand buffer, 2 μl of 0.1 M DTT and 1 μl of Moloney Murine Leukemia Virus Reverse Transcriptase (Invitrogen) and RNase free water to have a final volume of 20 μl qPCR was performed in a CFX 384 Thermal Cycler (Bio-Rad) with a 10 μl reaction mix containing 3 μl 100x diluted cDNA, forward and reverse primers (Supplementary Table S1) and 5 μl iQ SYBR Green SuperMix (170-8886, Bio-Rad). Amplification efficiencies of all primers were verified using a dilution series with a standard curve method. Ct values were obtained using the following thermal protocol: 95°C (3 min), 40 cycles of 95°C (20 s), 60°C (20 s), and 72°C (30 s), 95°C (1 min) and 65°C (1 min), concluded with a melting curve measurement. The commercial primer B2M (PrimerDesign) was used as a housekeeping gene, as determined being the most stable. Ct values were determined by thresholding at 200 relative fluorescence units within the exponential part of the curve. Ct values were normalized for the housekeeping gene by relative quantification (Livak and Schmittgen, 2001).

### Statistical analyses

The qPCR data from the static conditions were normalized to the geometric mean of the synthetic control group to obtain relative expression values. The data were presented as individual data points representing different experiments (*n =* 4–9), and their median. The normal distribution of data was checked with the Shapiro Wilk test. Since the data was not normally distributed, the non-parametric Kruskal-Wallis test with a Dunn’s multiple comparison test was performed to identify statistically significant differences between synthetic control and contractile control expressions, and within synthetic and contractile cell groups. Differences were considered statistically significant when *p < 0.05*.

The qPCR data from strain conditions were normalized to their corresponding synthetic or contractile static control groups. The normalized data were matched with strain data and presented as individual points representing different experiments. A non-parametric Spearman rank correlation test was used to measure the degree of correlation between mRNA expression and strain. *p*-values correspond to the significance level of the Spearman correlation coefficient and were considered statistically significant when *p < 0.05*.

## Results

### Jagged1-mediated Notch activation increases contractile characteristics of vascular smooth muscle cells

**FIGURE 2:**
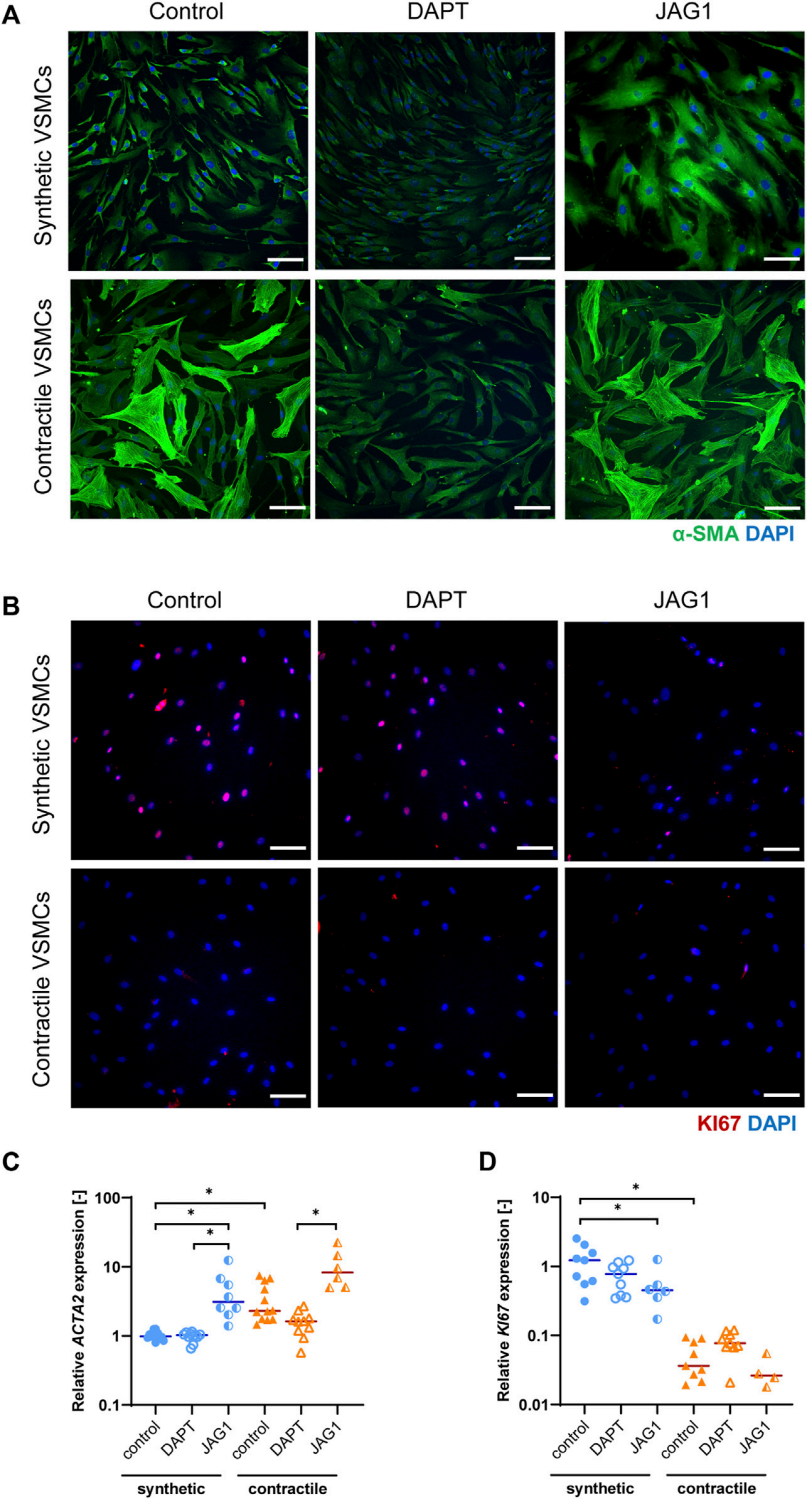
Characterization of synthetic and contractile VSMCs, and the impact of Notch signal inhibition and activation on VSMC phenotype. Representative IF images of **(A)** αSMA in green and nuclei in blue, **(B)** KI67 in red and nuclei in blue for control, DAPT-treated and JAG1-induced synthetic and contractile VSMCs (scale bar: 100 μm). Gene expression of phenotype markers **(C)** *ACTA2* and **(D)** *KI67* normalized to the synthetic control condition (*p < 0.05).

To characterize the phenotypes of synthetic and contractile human coronary artery smooth muscle cells, we performed IF staining and qPCR analyses for phenotypic markers. Contractile VSMCs demonstrated increased levels and a more fibrous organization of contractile marker αSMA compared to synthetic VSMCs (Figure 2A). In line with this, contractile VSMCs demonstrated increased mRNA expression of *ACTA2* (Figure 2C). Contractile VSMCs were also less proliferative than synthetic VSMCs, which was evident from decreased mRNA and protein expression of *KI67* (Figures 2B,C).

To elucidate the impact of Notch on the VSMC phenotype, we inhibited signaling by DAPT, and activated Notch signaling by seeding the VSMCs on immobilized Jagged1 ligands. The inhibition and activation of Notch signaling with this system was verified and shown in our previous work (Loerakker et al., 2018). We observed that, in comparison to the control group, the fibrous organization of αSMA was lost in contractile VSMCs after DAPT treatment (Figure 2A). In addition, *ACTA2* expression was generally lower in DAPT-treated contractile VSMCs compared to the control group, although these differences did not reach statistical significance (Figure 2C). *KI67* mRNA and protein expression did not change significantly in DAPT-treated contractile VSMCs (Figures 2B,D). Furthermore, DAPT-treatment hardly affected the *ACTA2* and *KI67* expression, and αSMA and KI67 staining in synthetic cells (Figures 2A–D). In contrast, the activation of Notch signaling increased the contractile features of both synthetic and contractile VSMCs compared to the DAPT-treated groups, as demonstrated by the αSMA staining and *ACTA2* expression (Figures 2A,C). Moreover, Notch activation significantly decreased the proliferative feature of synthetic VSMCs compared to control groups (Figures 2B,D).

In summary, these observations show that Jagged1-mediated Notch activation increases the contractile characteristics of VSMCs. DAPT-mediated inhibition of Notch signaling reduced, although not significantly, the expression of contractility proteins in contractile VSMCs, in line with the role of Notch in maintaining the contractile properties of VSMCs (Baeten and Lilly, 2017).

### Synthetic and contractile cells exhibit differential expression of Notch ligands and receptors

**FIGURE 3:**
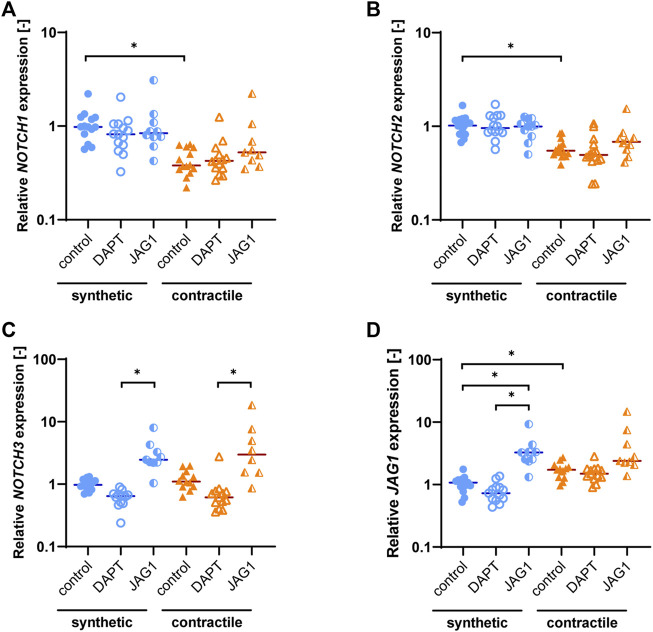
Notch pathway gene expression in synthetic and contractile VSMCs, and the impact of Notch signal inhibition and activation on Notch pathway genes. Gene expression of Notch signaling components **(A)** *NOTCH1*, **(B)** *NOTCH2*, **(C)** *NOTCH3*, **(D)** *JAG1*, normalized to the synthetic control condition (*p < 0.05).

As the Notch signaling pathway has been shown to regulate VSMC phenotype (Domenga et al., 2004; Doi et al., 2006; High et al., 2008), we assessed the expression of Notch pathway genes in synthetic and contractile VSMCs. Contractile VSMCs showed an increased expression of *JAG1* and a reduced expression of *NOTCH1* and *NOTCH2* compared to synthetic VSMCs (Figures 3A,B). The expression levels of *NOTCH3* were similar in both phenotypes (Figure 3C). The inhibition of Notch signaling generally lowered the expression of *NOTCH3* and *JAG1*, although not significantly, and the activation of Notch signaling significantly increased the expression of *NOTCH3* in both synthetic and contractile VSMCs, and *JAG1* in synthetic cells (Figures 3C,D). Expression of *NOTCH1* and *NOTCH2* was unaffected by the inhibition and activation of Notch signaling (Figures 3A,B).

### Jagged1-mediated Notch activation increases extracellular matrix expression in synthetic and contractile vascular smooth muscle cells

**FIGURE 4:**
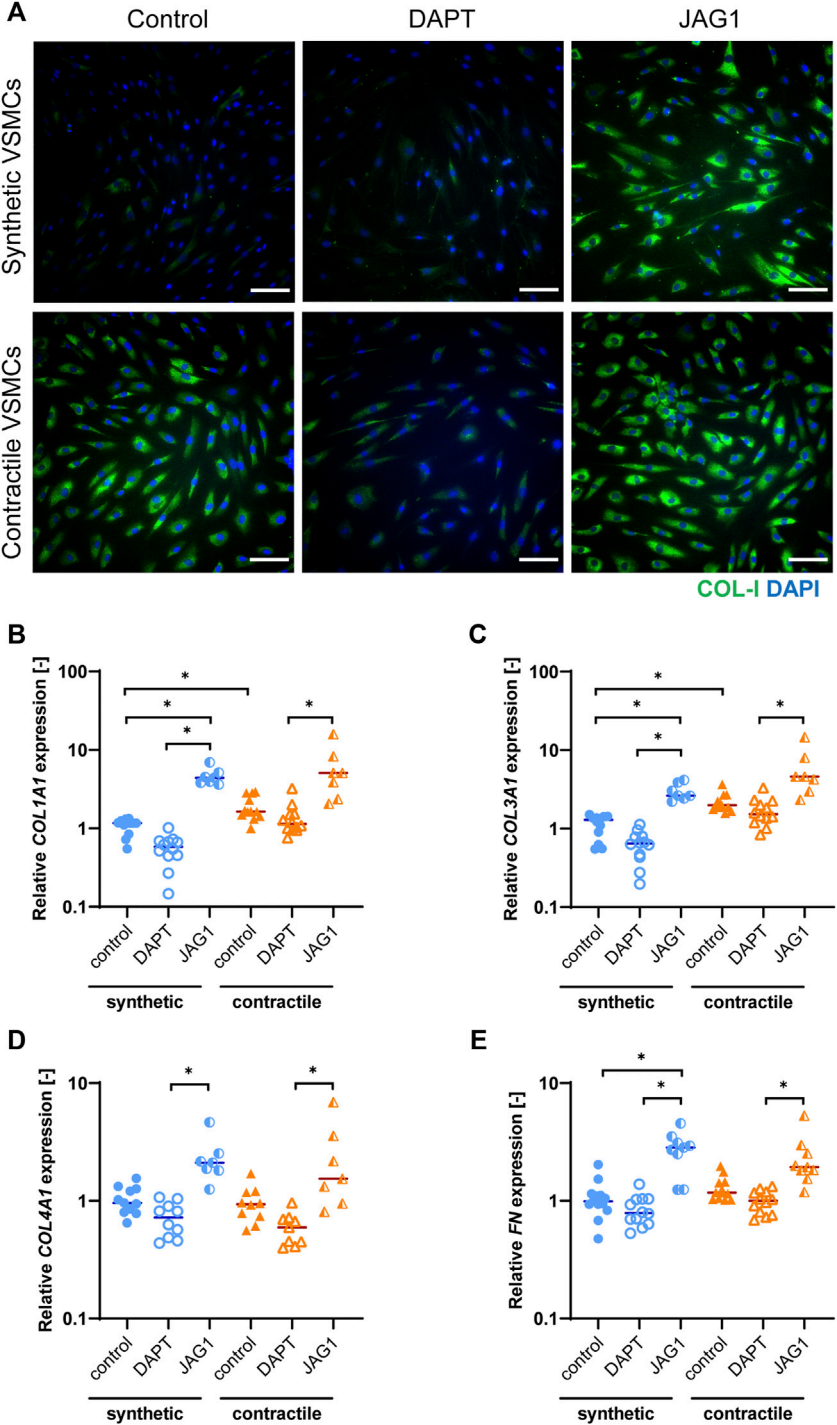
ECM production in synthetic and contractile VSMCs, and the impact of Notch signal inhibition and activation on ECM production. **(A)** Representative IF images of collagen I in green and nuclei in blue for control, DAPT-treated and JAG1-induced synthetic and contractile VSMCs (scale bar: 100 μm). Gene expression of ECM components **(B)** *COL1A1*, **(C)** *COL3A1*, **(D)** *COL4A1*, **(E)** *FN*, normalized to the synthetic control condition (*p < 0.05).

To characterize the extracellular matrix (ECM) production of synthetic and contractile VSMCs, and to assess the impact of Notch signaling on ECM production, we analyzed the expression of ECM components collagen I, III, IV and fibronectin in control, DAPT-treated and Jagged1-induced synthetic and contractile VSMCs. Contractile VSMCs demonstrated more collagen I staining compared to synthetic VSMCs (Figure 4A). Furthermore, contractile VSMCs expressed higher levels of *COL1A1* and *COL3A1* than synthetic VSMCs (Figures 4B,C), and no difference between synthetic and contractile VSMCs was observed with regard to *COL4A1* and *FN* expression (Figures 4D,E). DAPT-mediated Notch inhibition generally resulted in lower expression levels of all investigated ECM components compared to control groups in both synthetic and contractile VSMCs, although these differences were not statistically significant (Figures 4B–E). In contrast, Jagged1-mediated Notch activation increased the expression of all investigated ECM components in both synthetic and contractile VSMCs (Figures 4B–E). This effect was more pronounced in synthetic VSMCs (Figure 4).

### Strain induces a loss of contractile features in contractile vascular smooth muscle cells

To determine the effect of strain on VSMC phenotype, we mechanically stretched synthetic and contractile VSMCs. We used different strain levels, and quantified the applied strain for each sample. We performed IF staining and qPCR analyses of phenotypic markers on static and stretched samples. In addition, we assessed the correlation between the quantified strain levels and gene expression results using a non-parametric Spearman correlation coefficient to determine the statistical significance of the correlations.

**FIGURE 5:**
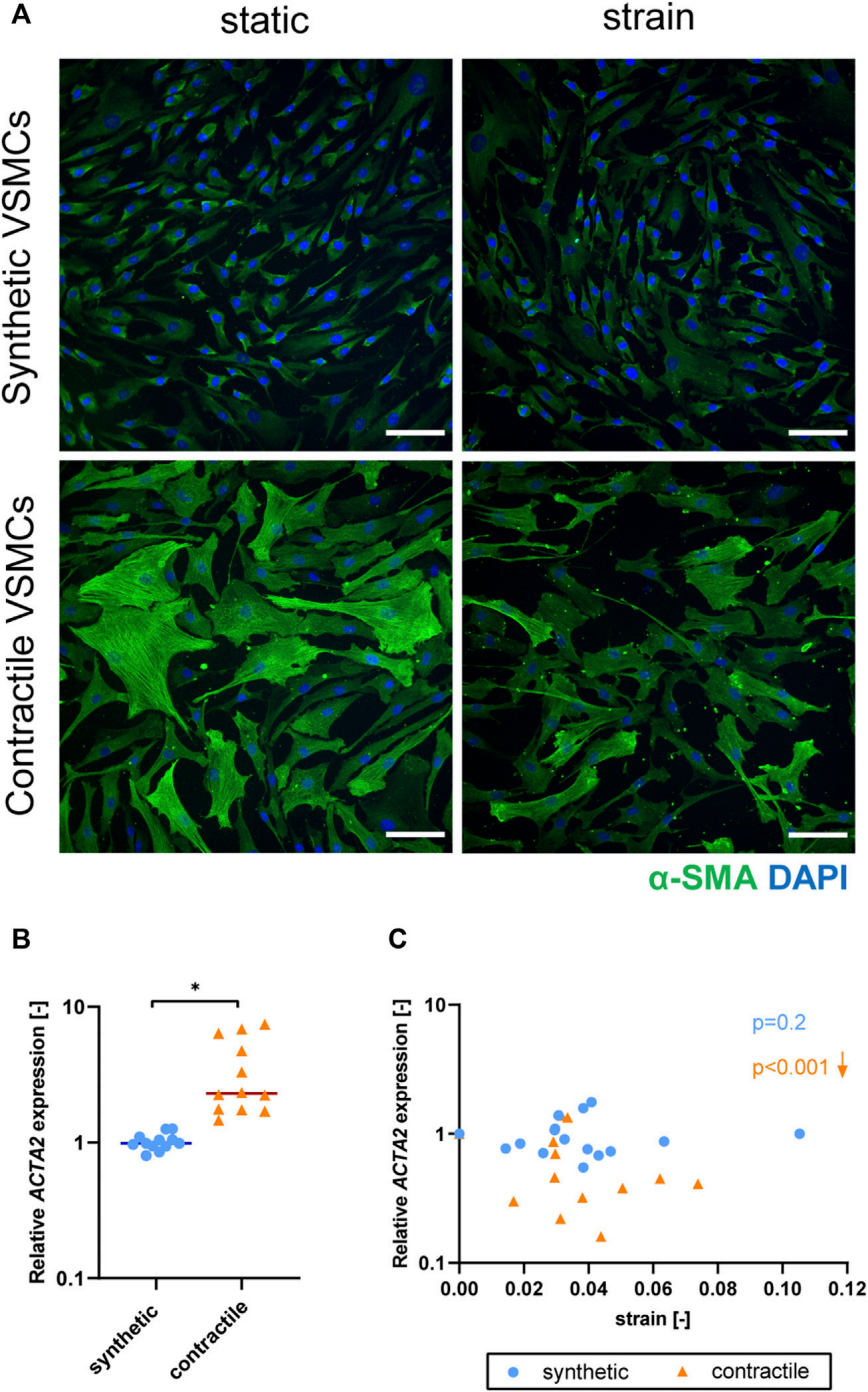
The effect of strain on VSMC phenotype. **(A)** Representative IF images of αSMA (green) and nuclei (blue) in synthetic and contractile VSMCs, in the absence of strain (static) and upon the application of 0.04 (4%) strain for 48 h (scale bar: 100 μm). Gene expression of phenotype marker *ACTA2* for synthetic (blue circles) and contractile (orange triangles) VSMCs **(B)** in the absence of strain (normalized to the synthetic control) and **(C)** as a function of strain (each data point is normalized to its corresponding static control expression). *p* values show the significance level of non-parametric Spearman correlation coefficient. The arrows next to significant correlations (*p < 0.05*) demonstrate an up or downregulation in gene expression with applied strain levels.

Upon the application of strain, contractile VSMCs switched towards a more synthetic phenotype in terms of decreased levels of αSMA staining and *ACTA2* expression, while the contractile features of synthetic VSMCs were not significantly affected by strain (Figure 5). The decrease in *ACTA2* expression in contractile VSMCs upon the application of strain correlated with the applied strain levels while no significant correlation with strain was detected in the expression levels of synthetic VSMCs (Figure 5C).

### Expression of *NOTCH3* and *JAG1* decreases with strain in both phenotypes

**FIGURE 6:**
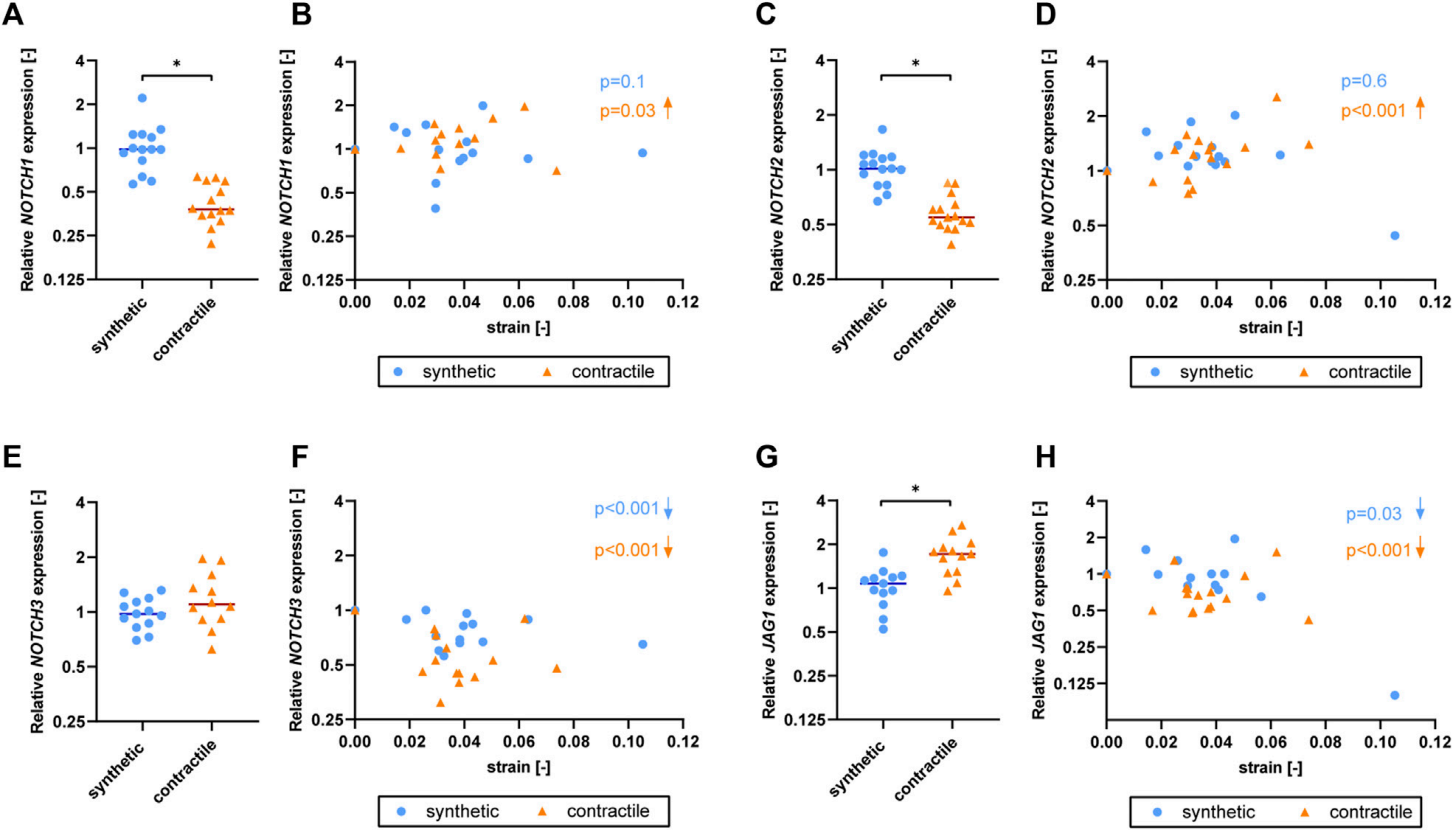
The effect of strain on Notch pathway genes for synthetic and contractile VSMCs. Gene expression of Notch signaling components **(A,B)** *NOTCH1*, **(C,D)** *NOTCH2*, **(E,F)** *NOTCH3* and **(G,H)** *JAG1* for synthetic (blue circles) and contractile (orange triangles) VSMCs **(A,C,E,G)** in the absence of strain (normalized to the synthetic control) and **(B,D,F,H)** as a function of strain (each data point is normalized to its corresponding static control expression). *p* values show the significance level of non-parametric Spearman correlation coefficient. The arrows next to significant correlations (*p < 0.05*) demonstrate an up or downregulation in gene expression with applied strain levels.

To understand the effect of strain on Notch signaling components, we assessed the expression of Notch pathway genes in synthetic and contractile VSMCs upon the application of strain (Figure 6). *NOTCH1* and *NOTCH2* expressions were responsive to strain only in contractile VSMCs (Figures 6B,D). *NOTCH3* and *JAG1* expressions were affected by strain in both phenotypes of VSMCs, where the contractile VSMCs showed a stronger effect compared to the synthetic VSMCs (Figures 6F,H). In contractile VSMCs, *NOTCH1* and *NOTCH2* expressions increased upon the application of strain (Figures 6B,D). *NOTCH3* and *JAG1* expressions decreased with increasing strain levels in both phenotypes, and this decrease was more prominent in the contractile VSMCs (Figures 6F,H).

### Extracellular matrix expression is affected by strain only in synthetic vascular smooth muscle cells

**FIGURE 7:**
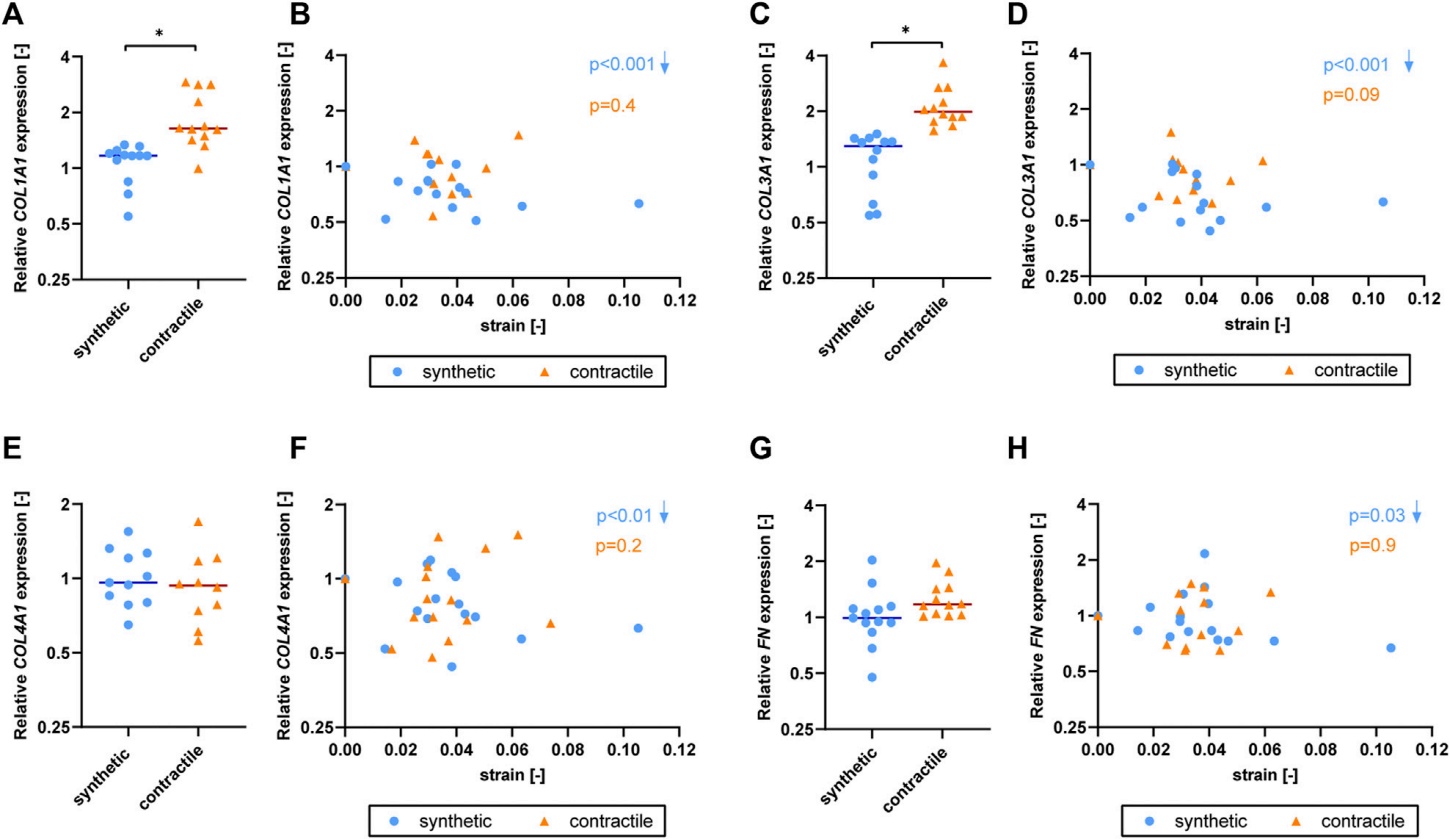
The effect of strain on ECM for synthetic and contractile VSMCs. Gene expression of ECM components **(A,B)** *COL1A1*, **(C,D)** *COL3A1*, **(E,F)** *COL4A1* and **(G,H)** *FN* for synthetic (blue circles) and contractile (orange triangles) VSMCs **(A,C,E,G)** in the absence of strain (normalized to the synthetic control) and **(B,D,F,H)** as a function of strain (each data point is normalized to its corresponding static control expression). *p* values show the significance level of non-parametric Spearman correlation coefficient. The arrows next to significant correlations (*p < 0.05*) demonstrate an up or downregulation in gene expression with applied strain levels.

To determine the effect of strain on ECM production of synthetic and contractile VSMCs, we measured the expression of ECM components upon the application of strain and correlated quantified strain levels with gene expression results (Figure 7). We observed that only the synthetic VSMCs showed a response to strain in terms of ECM expression. Specifically, *COL1A1, COL3A1, COL4A1,* and *FN* expression in synthetic VSMCs decreased upon the application of strain, while no significant correlation with strain was detected in the expression levels of contractile VSMCs (Figures 7B,D,F,H).

### Jagged1-mediated Notch activation partially rescues strain-mediated phenotypic switching in contractile vascular smooth muscle cells

To elucidate the role of Notch signaling in strain-mediated phenotypic changes of VSMC, we inhibited Notch signaling by DAPT, and activated Notch signaling by seeding the VSMCs on immobilized Jagged1 ligands during the process of mechanical stretching. We compared stretched samples of control, DAPT-treated and Jagged1-induced synthetic and contractile VSMCs by means of IF staining and qPCR for the changes in contractile features. We quantified the applied strain per sample to determine gene expression as a function of strain.

**FIGURE 8:**
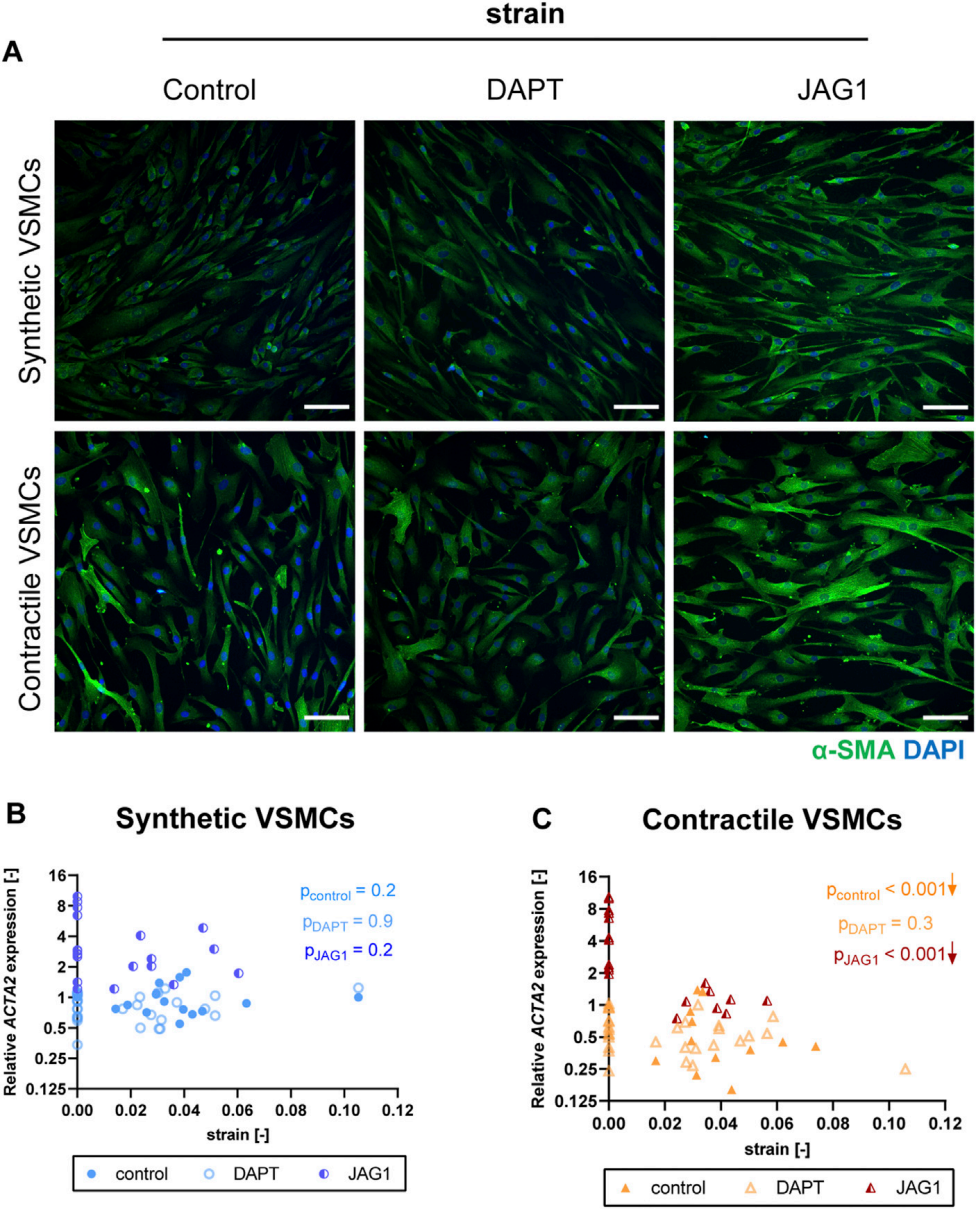
The impact of Notch signal inhibition and activation on strain-mediated changes in VSMC phenotype. **(A)** Representative IF images of αSMA (green) and nuclei (blue) for control, DAPT-treated and JAG1-induced synthetic and contractile VSMCs upon the application of 0.04 (4%) strain for 48 h (scale bar: 100 µm). Gene expression of phenotype marker *ACTA2* for control, DAPT-treated and JAG1-induced **(B)** synthetic VSMCs (blue circles) and **(C)** contractile VSMCs (orange triangles) as a function of strain (normalized to static control expression). *p* values show the significance level of non-parametric Spearman correlation coefficient. The arrows next to significant correlations (*p < 0.05*) demonstrate an up or downregulation in gene expression with applied strain levels.

The contractile features of synthetic VSMCs were hardly affected by the inhibition of Notch signaling during the application of strain. In addition, contractile VSMCs, which switched towards a synthetic phenotype in terms of αSMA content and organization with strain, did not show a further change in their contractile features when Notch signaling was inhibited (Figure 8A). In addition, a similar *ACTA2* expression was detected between control and DAPT-treated samples both in synthetic and contractile VSMC groups (Figures 8B,C). On the other hand, Notch activation with immobilized Jagged1 ligands increased the expression of contractile markers, and partially rescued the contractile features of contractile VSMCS upon strain application (Figure 8A). Moreover, *ACTA2* expression in Notch-activated synthetic and contractile VSMCs was generally higher than their corresponding control and DAPT-treated groups, even upon the application of strain (Figure 8C). This shows the important role of Notch signaling in strain-mediated phenotypic changes.

### Jagged1-mediated Notch activation during stretching upregulates *NOTCH3* and *JAG1* expression in both synthetic and contractile vascular smooth muscle cells

**FIGURE 9:**
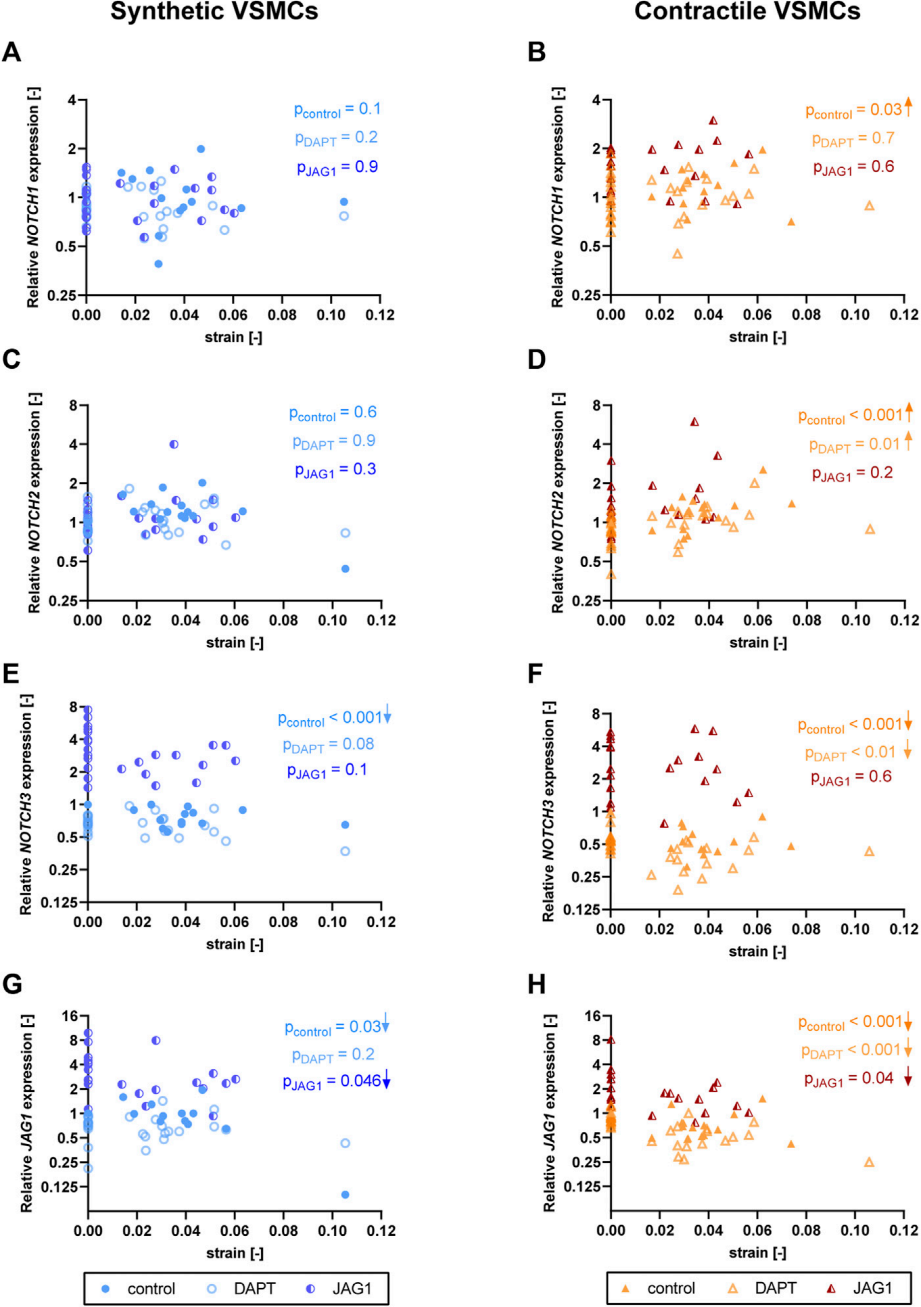
The impact of Notch signal inhibition and activation on strain-mediated changes in Notch pathway genes. Gene expression of Notch signaling components **(A,B)** *NOTCH1*, **(C,D)** *NOTCH2*, **(E,F)** *NOTCH3* and **(G,H)** *JAG1* for control, DAPT-treated and JAG1-induced **(A,C,E,G)** synthetic VSMCs (blue circles) and **(B,D,F,H)** contractile VSMCs (orange triangles) as a function of strain (normalized to static control expression). *p* values show the significance level of non-parametric Spearman correlation coefficient. The arrows next to significant correlations (*p < 0.05*) demonstrate an up or downregulation in gene expression with applied strain levels.

To investigate the role of Notch signaling in strain-mediated changes in Notch pathway genes, we assessed the *NOTCH1, NOTCH2, NOTCH3,* and *JAG1* levels in synthetic and contractile VSMCs, which were DAPT-treated and Jagged1-induced during mechanical stretching (Figure 9). Expression of *NOTCH1* and *NOTCH2* was only marginally affected by DAPT-mediated Notch inhibition and Jagged1-mediated Notch activation during stretching in both phenotypes (Figures 9A–D). On the other hand, Notch activation during stretching upregulated expression of *NOTCH3* and *JAG1* in both synthetic and contractile VSMCs (Figures 9E–H). Moreover, *NOTCH3* expression, which was downregulated by strain in control samples, did not decrease with strain upon Notch activation in synthetic and contractile VSMCs (Figures 9E,F), in line with the observation of the partial rescue of contractile features in contractile VSMCs with Notch activation during stretching. Interestingly, *JAG1* expression still decreased with strain upon Notch activation in synthetic and contractile VSMCs (Figures 9G,H), which correlated with the decrease in *ACTA2* levels with strain in Notch-activated contractile VSMCs (Figure 8C).

### Jagged1-mediated Notch activation during stretching increases extracellular matrix expression in both synthetic and contractile vascular smooth muscle cells

**FIGURE 10:**
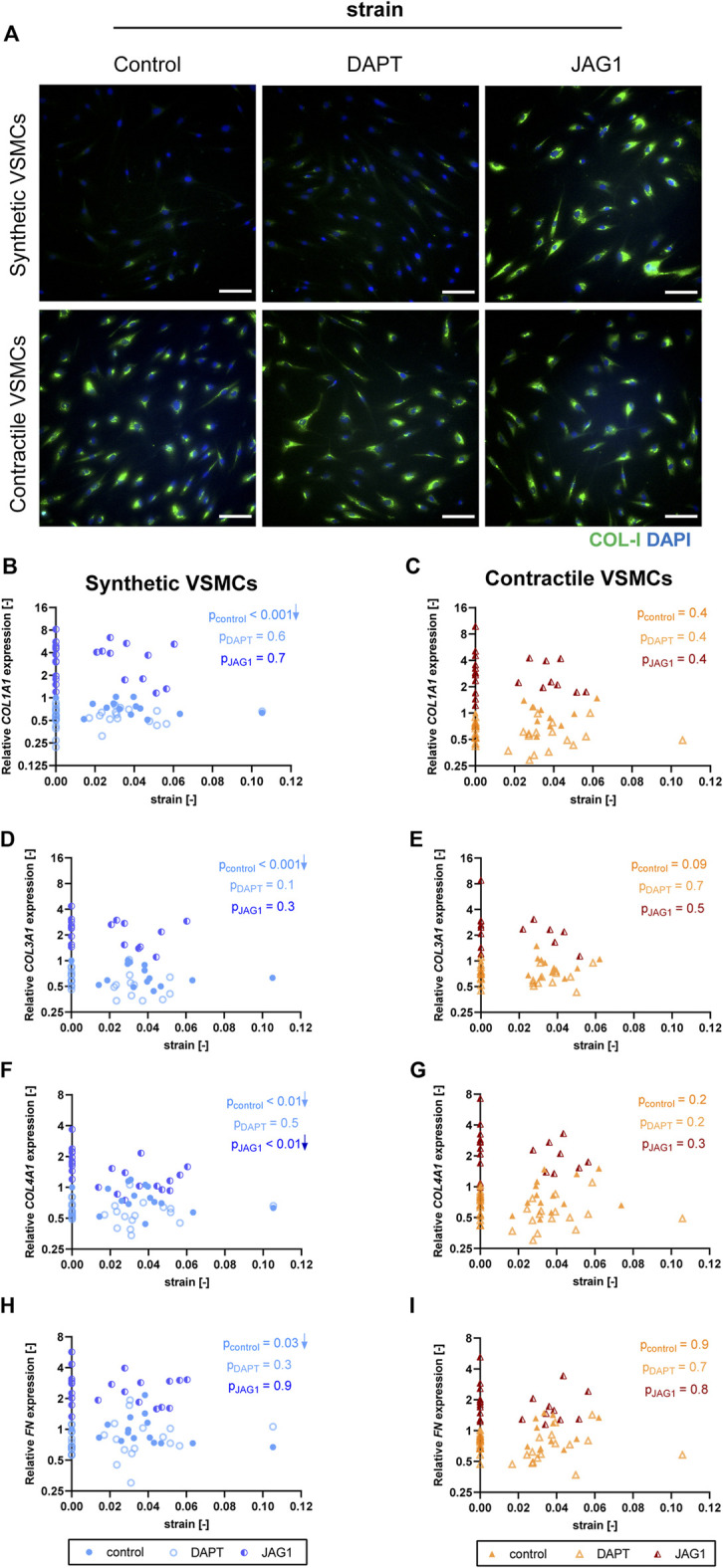
The impact of Notch signal inhibition and activation on strain-mediated changes in ECM production. **(A)** Representative IF images of collagen I (green) and nuclei (blue) for control, DAPT-treated and JAG1-induced synthetic and contractile VSMCs upon the application of 0.04 (4%) strain for 48 h (scale bar: 100 µm). Gene expression of ECM components **(B,C)** *COL1A1*, **(D,E)** *COL3A1*, **(F,G)** *COL4A1* and **(H,I)** *FN* for control, DAPT-treated and JAG1-induced **(B,D,F,H)** synthetic VSMCs (blue circles) and **(C,E,G,I)** contractile VSMCs (orange triangles) as a function of strain (normalized to static control expression). *p* values show the significance level of non-parametric Spearman correlation coefficient. The arrows next to significant correlations (*p < 0.05*) demonstrate an up or downregulation in gene expression with applied strain levels.

To assess the role of Notch signaling in strain-mediated changes of ECM production, we measured the expressions of ECM components in synthetic and contractile VSMCs, which were DAPT-treated and Jagged1-induced during mechanical stretching. DAPT-mediated Notch inhibition during stretching hardly affected the ECM expression of synthetic and contractile VSMCs compared to their corresponding control levels (Figure 10). On the other hand, Notch activation elevated the expression levels of all investigated ECM proteins in both phenotypes (Figure 10). In addition, the observed strain-dependence of ECM expression, particularly *COL1A1*, *COL3A1*, and *FN* levels, in synthetic VSMCs was absent in the Notch-activated synthetic VSMCs (Figures 10B,D,H).

## Discussion

Unravelling the mechanobiological mechanisms regulating VSMC fate is necessary to better understand vascular development and pathologies, and to direct growth and remodeling processes in vascular tissue engineering. Mechano-regulated cell signaling pathways could explain the link between mechanical strain and VSMC behavior regulating the growth and remodeling. Here, we investigated the role of mechanosensitive Notch signaling in regulating strain-mediated changes in VSMC phenotype. Our data demonstrate that strain decreases the expression of Notch components and induces a loss of contractile features. The activation of Notch signaling partially rescues the strain-mediated changes in contractile features, suggesting that the mechanosensitive Notch pathway can directly control the phenotypic features of VSMCs.

Our data show that Notch signaling promotes and maintains the contractile features of VSMCs, in line with previous findings (Domenga et al., 2004; Wang Q. et al., 2012; Hoglund and Majesky, 2012; Lin and Lilly, 2014a; Liu N. et al., 2015). Interestingly, in our study contractile cells expressed more collagen-I and collagen-III compared to synthetic VSMCs. This is inconsistent with the common definition of synthetic versus contractile VSMCs, based on previous studies performed *in vivo* that have classified synthetic VSMCs as cells with extensive ECM synthesis during vascular development and upon vascular injury, and contractile VSMCs as cells typically showing a very low rate of ECM synthesis but high contractility (Owens et al., 2004; Beamish et al., 2010). On the other hand, our data is consistent with other studies performed *in vitro*, showing that VSMCs conditioned in low serum and heparin rich medium, used here to differentiate VSMCs towards the contractile phenotype, express higher levels of collagen (Majack and Bornstein, 1985; Liau and Chan, 1989). This suggests that different definitions of VSMC phenotypes might be necessary, going beyond the synthetic versus contractile paradigm (Li et al., 2020; Yap et al., 2021). Although it is accepted that these phenotypes are the extremes of a continuous spectrum and that VSMCs might adopt a phenotype within this continuum (Stegemann et al., 2005; Rensen et al., 2007; Beamish et al., 2010; Estrada et al., 2021), our data suggest that these phenotypes are not exclusive, and that VSMCs with contractile features can exhibit some of the synthetic characteristics, at least *in vitro*. It is, therefore, possible that synthetic and contractile VSMCs cultured *in vitro* do not represent the full extremes of the phenotypic spectrum, but still show certain main characteristics of their phenotypes. Despite these limitations, our *in vitro* differentiation method upregulates the contractile features of VSMCs, irrespective of the common definition of VSMC phenotype.

Differences in terms of definition of VSMC phenotypes could also explain inconsistencies between previous studies analyzing the effects of mechanical cues on VSMCs. Cyclic strain is known to regulate the phenotypic switching of VSMCs (Jensen et al., 2021), but *in vitro* cyclic strain has been reported to both up and downregulate the expression of contractile markers in VSMCs (Reusch et al., 1996; Tock et al., 2003; Butcher et al., 2006; Hu et al., 2014; Yao et al., 2014; Rodríguez et al., 2015; Wan et al., 2015; Wang et al., 2018). *In vivo* studies have shown that, as a response to alterations in hemodynamic conditions, contractile VSMCs switch to a more synthetic phenotype (Owens et al., 2004). Our *in vitro* results are in agreement with these *in vivo* findings; in particular, we showed that cyclic equibiaxial strain downregulates the contractile features of contractile VSMCs *in vitro*, while synthetic VSMCs were not significantly affected. Thereby, this suggests a possible reason for the inconsistencies of previous *in vitro* studies where different VSMC phenotypes may have been employed before stretching. In addition, the diversity in cell sources and surface coatings, as well as differences in magnitude, direction, and duration of the applied strain could also explain the variations in outcome (Reusch et al., 1996; Tock et al., 2003; Butcher et al., 2006; Hu et al., 2014; Yao et al., 2014; Rodríguez et al., 2015; Wan et al., 2015; Wang et al., 2018). A more systematic investigation of how each of these parameters affects the role of stretch on regulating the VSMC phenotype would therefore be necessary.

Our data also suggest that Notch plays a role in regulating ECM production of VSMCs. In fact, Jagged1-mediated Notch activation increased the expression of collagen-I, III, IV and fibronectin (Figure 4). Activation of Notch also upregulated the expression of ECM genes during stretching, suggesting that Notch might have an important role in ECM homeostasis during mechanical stress. Previous studies have shown that ECM proteins can affect Notch signaling directly by interacting with Notch receptors/ligands, or indirectly by up or downregulating Notch receptors/ligands (LaFoya et al., 2016; Condorelli et al., 2021). However, to our knowledge, the role of Notch in regulating ECM production in VSMCs had not been explored yet. Future studies should further investigate this bi-directional feedback between Notch and ECM, as this interaction may be of major importance when considering Notch-mediated ECM remodeling of vascular tissues.

Our results further showed that strain downregulated ECM expression in synthetic VSMCs, but not in contractile VSMCs (Figure 7). Previous studies showed that strain increases ECM production in VSMCs, but this increased synthesis is typically observed at higher strain levels (more than 10%) (Stanley et al., 2000; Liu X. et al., 2015) and longer exposure time (4–5 days) compared to our experiments (Sumpio et al., 1988; O’Callaghan and Williams, 2000). Additionally, a downregulation or no change in collagen production as a short-term effect of cyclic strain has also been detected in tissue engineered constructs seeded with vascular-derived cells (Van Geemen et al., 2013). Thus, our results confirm a different short-term response of synthetic versus contractile VSMCs to strain in terms of ECM synthesis. Moreover, since we only applied strain for a period of 2 days, because the DAPT treatment for longer periods significantly affected the cell survival, our results on the expression of ECM components may have limited value with regard to representing the strain-dependent ECM production of VSMCs at larger strain magnitudes and durations.

The mechanosensitive properties of Notch seem to be particularly important in the cardiovascular system, especially its regulation by strain (Morrow et al., 2005; Loerakker et al., 2018; Karakaya et al., 2022). In agreement with previous findings (Morrow et al., 2005; Loerakker et al., 2018), our results show that *NOTCH3* and *JAG1* expressions are downregulated by strain. In addition, contractile VSMCs showed a stronger response compared to synthetic VSMCs. This stronger response is in line with the fact that Notch activity is higher in contractile VSMCs and, as a result, it can be downregulated more by strain to regulate the switch towards a synthetic fate. This therefore suggests that the strain-dependence of Notch gene expression may depend on the levels of Notch activity in a specific VSMC phenotype. On the other hand, the strain-dependence of Notch expression did not depend on the strain magnitude (Figures 6F,H). In fact, relatively large changes in expression occurred already at small strain magnitudes, and differences between different strain magnitudes were relatively minor compared to differences with respect to the static condition, as in Loerakker et al., 2018. On the other hand, there may be further mechanosensitive post-translational modifications that can alter the Notch response at a protein level. Nevertheless, although this conclusion should be further verified by other analysis methods, our data suggest a switch-like dependency of Notch to cyclic strain, rather than a response that is strongly dependent on the strain magnitude.

**FIGURE 11:**
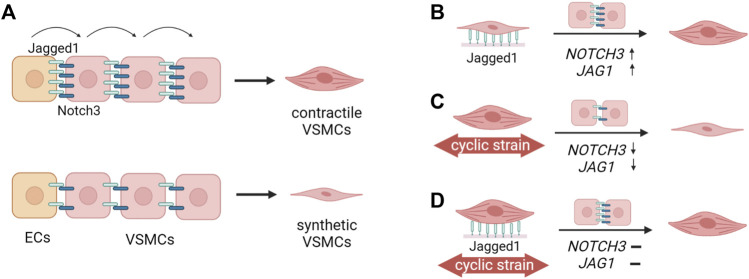
Schematic summary of the findings. **(A)** Jagged1-mediated Notch signaling initiated by the endothelial cells (ECs) propagates through the vessel wall (Hoglund and Majesky, 2012; Manderfield et al., 2012) and determines the differentiation of vascular smooth muscle cells (VSMCs) towards the contractile phenotype (Domenga et al., 2004; Doi et al., 2006; High et al., 2007). High Jagged1-Notch3 activity leads to the contractile phenotype of VSMCs, and low Jagged1-Notch activity is associated with the synthetic phenotype of VSMCs. **(B)** In this study, we showed that Jagged1-induction upregulated *NOTCH3* and *JAG1* expression, and increased the contractile features of VSMCs. This shows that Notch signaling directly influences the VSMC phenotype. **(C)** The application of strain to contractile VSMCs downregulates *NOTCH3* and *JAG1* expressions, and switches contractile VSMCs towards a more synthetic phenotype **(D)** Jagged1-induction during the application of strain partially rescues the contractile phenotype of VSMCs. Thus, Notch signaling plays an important role in strain-mediated phenotypic switching of VSMCs (The figure was created with BioRender.com).

Our data indicate that Notch can independently increase or maintain the contractile features of VSMCs, suggesting that the observed changes in Notch signaling upon strain are at least a partial cause of the phenotypic switching of VSMC in response to strain (Figure 11). In fact, during stretching, Notch activation *via* immobilized Jagged1 substantially upregulated the expression of *NOTCH3* and *JAG1* in both synthetic and contractile VSMCs, and induced a partial rescue of contractile features. Yet, *JAG1* and *ACTA2* expression in Notch-activated VSMCs upon strain was still lower compared to statically cultured contractile VSMCs. The concentration of immobilized Jagged1 ligands in our experiments may have been just sufficient to partially rescue the contractile phenotype, but not able to fully overrule the effects of strain on Notch signaling. It is good to mention that our results on Notch are limited to gene expression analysis. Other experimental methods such as protein analyses and reporter assays would give more information on the activity of Notch at protein level, and complement the qPCR results presented in this study. Nevertheless, our data show that Notch has a direct effect on strain-mediated phenotypic changes of VSMCs, and this provides a starting point for additional analyses to obtain a more complete understanding.

*NOTCH1* and *NOTCH2* were not responsive to strain in synthetic cells, in agreement with Loerakker et al., 2018; however, they were upregulated by strain in contractile VSMCs. The increase of *NOTCH1* and *NOTCH2* in contractile VSMCs as a response to strain could be related to the pathophysiological roles of Notch1 and Notch2 in regulating arterial remodeling (Li et al., 2009; Boucher et al., 2013; Redmond et al., 2014). The levels of Notch1 and Notch2 increase in VSMCs after vascular injury and promote neointimal formation (Li et al., 2009; Boucher et al., 2013). Our data thus show Notch receptor-specific responses to mechanical stimuli. The differential up and downregulation of Notch receptor expression by strain could be related to their functions. Notably, Notch2 and Notch3 have opposing effects on cell proliferation and survival in VSMCs (Baeten and Lilly, 2015). Therefore, a different regulation of these two receptors by strain is not unexpected and might serve as a mechanism to fine-tune VSMC behavior in mechanically changing environments.

Taken together, our results demonstrate the importance of Notch signaling in regulating strain-mediated phenotypic switching of VSMCs. Our findings suggest that VSMC fate can be (partly) controlled *via* leveraging the interplay between Notch signaling and strain, and highlight Notch signaling as a potential target in vascular therapy and regenerative medicine. These findings might help the design of new bioactive materials for *in situ* tissue engineering approaches, where tissue and ECM homeostasis have to be restored under the physiological stress environment to restore functional tissues. Yet, the dose-responsiveness of Notch signaling to strain in terms of controlling the VSMC fate and the distinct roles of the different receptors in regulating VSMC phenotype and vascular remodeling should be further investigated to fully understand the opportunities and limitations of regulating VSMC phenotype *via* the mechanosensitive Notch pathway.

## Data Availability

All data and original codes for analyses have been deposited at 4TU.ResearchData, and are publicly available at https://doi.org/10.4121/20343000.v1.
