# Supplementary material for: Notch signaling regulates strain-mediated phenotypic switching of vascular smooth muscle cells
Source: Front Cell Dev Biol. 2022 Aug 12;10:910503. doi: 10.3389/fcell.2022.910503 (PMC9412035; doi:10.3389/fcell.2022.910503)
Supplement: Supplementary file 1 [file DataSheet1.PDF]

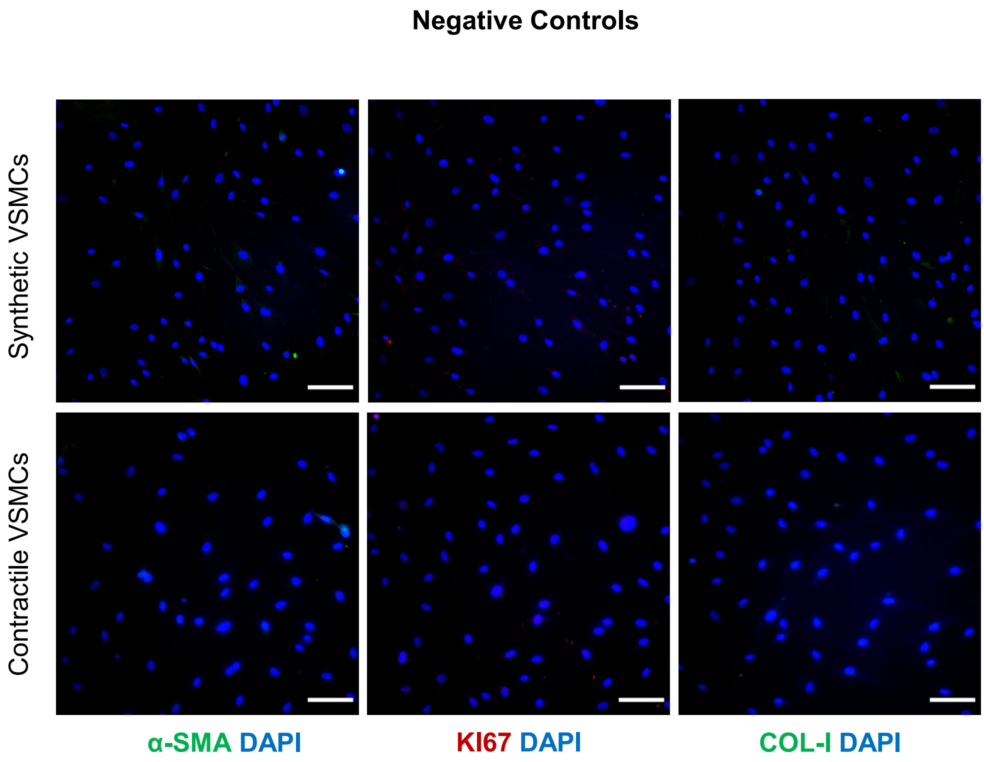

Supplementary Figure 1: Negative secondary antibody controls of IF staining for αSMA (green), KI67 (red) and collagen I (green) in synthetic and contractile VSMCs. Cell nuclei are stained with DAPI (blue) in all images (scale bar: 100 μm).

Supplementary Table 1: The list of human genes used for qPCR with their forward and reverse primer sequences and final concentrations in the reaction.

Gene	Strand	Sequence (5' → 3')	Concentration
B2M	Forward	PrimerDesign	1000nM
	Reverse	PrimerDesign	
ACTA2	Forward	CGTGTTGCCCCTGAAGAGCAT	1000nM
	Reverse	ACCGCCTGGATAGCCACATACA	
KI67	Forward	CTTTGGGTGCGACTTGACG	1000nM
	Reverse	GTCGACCCCGCTCCTTTT	
COL1A1	Forward	AATCACCTGCGTACAGAACGG	1000nM
	Reverse	TCGTCACAGATCACGTCATCG	
COL3A1	Forward	ATCTTGGTCAGTCCTATGC	1000nM
	Reverse	TGGAATTTCTGGGTTGGG	
COL4A1	Forward	ACTCTTTTGTGATGCACACCA	1000nM
	Reverse	AAGCTGTAAGCGTTTGCGTA	
FN1	Forward	AAGACCAGCAGAGGCATAAGG	1000nM
	Reverse	CACTCATCTCCAACGGCATAATG	
NOTCH1	Forward	CGGGGCTAACAAAGATATGC	500nM
	Reverse	CACCTTGGCGGTCTCGTA	
NOTCH2	Forward	AAGGAACCTGCTTTGATGACA	500nM
	Reverse	CAGGGAGCCAATACTGTCTGA	
NOTCH3	Forward	CCTAGTCCTGGCTCCGAAC	500nM
	Reverse	GAGCCGGTTGTCAATCTCC	
JAG1	Forward	AATGGCTACCGGTGTGTCTG	200nM
	Reverse	CCCATGGTGATGCAAGGTCT	
